# Trends in Dietary Intake of Folate, Vitamins B_6_, and B_12_ among Japanese Adults in Two Rural Communities from 1974 through 2001

**DOI:** 10.2188/jea.15.29

**Published:** 2005-05-10

**Authors:** Kaori Yoshino, Mieko Inagawa, Miyuki Oshima, Kimiko Yokota, Mitsumasa Umesawa, Ma Enbo, Kazumasa Yamagishi, Takeshi Tanigawa, Shinichi Sato, Takashi Shimamoto, Hiroyasu Iso

**Affiliations:** 1 Department of Public Health Medicine, Doctoral Program in Social and Environmental Medicine, Graduate School of Comprehensive Human Sciences, University of Tsukuba.; 2 Department of Public Health and Welfare, Kyowa Municipal Office.; 3 The Osaka Medical Center for Health Science and Promotion.

**Keywords:** Folic Acid, Vitamin B_6_, Vitamin B_12_, Food, trend

## Abstract

BACKGROUND: The 5th edition of the Japanese food composition table enables us to evaluate intakes of folate and vitamins B_6_ and B_12_, which are associated with risk of chronic diseases.

METHODS: We investigated long-term trends in dietary intake of those nutrients in two rural communities; Ikawa from 1974 through 2000, and Kyowa from 1982 through 2001. The 24-hour recall method was adopted. Intake of green tea interviewed from 1994 was used to examine food sources for these nutrients in the latest period, but not to evaluate long-term trends. Reduced intakes of nutrients due to cooking were not taken into account.

RESULTS: Age-adjusted mean folate intake increased by 30% in Ikawa between the 1970’s and 1980’s, and then leveled off to the latest survey, while that in Kyowa did not change throughout the survey periods. The increased folate intake was primarily due to green/yellow vegetables. Mean vitamin B_6_ intake did not change except that it increased for Ikawa women in the 1980’s and decreased for Kyowa men in the latest period. No secular trend was found for mean vitamin B_12_ intake. The largest source for folate intake was total vegetables (38-58% of total intake) and the second largest source was alcohol/beverages including green tea (11-24%). Fish/shellfish was the largest source for vitamins B_6_ (16-23%) and B_12_ (77-84%).

CONCLUSIONS: Dietary intakes of folate, vitamins B_6_ and B_12_ showed no notable long-term trend, except for an increased folate intake between the 1970’s and 1980’s due to an increased intake of green/yellow vegetables.

Folate, vitamins B_6_ and B_12_ act as coenzymes for homocysteine metabolism.^1^^,^^2^ Blood homocysteine levels are increased with light-to-moderate deficiency of these nutrients, in particular folate and vitamin B_12_,^3^^-^^6^ which has been recognized as a risk factor for coronary heart disease and ischemic stroke.^7^^-^^11^ High homocysteine concentrations are also associated with neural tube defects,^12^ hypothyroidism, hyperthyroidism,^13^ osteoporosis,^14^^,^^15^ Alzheimer disease,^16^ and Parkinson’s disease.^17^ Low folate intake is associated with risk of colon cancer.^18^^,^^19^ Based on previous studies, the recommended dietary allowance (RDA) for folate was determined as 400 µg/day for adults in the United States in 1997.^20^^-^^22^ In addition, folate-fortified cereals appeared in the market following to the recommendation issued in 1998 by the Federal Department of Agriculture.^23^^-^^25^ Depending on age and sex, the RDA for vitamin B_6_ is 1.3 to 1.7 mg/day, and that of vitamin B_12_ is 2.4 µg/day.^20^^-^^22^ In Japan, the RDA for folate is 200 µg/day and double that in pregnant women, 1.6 mg/day for vitamin B_6_ in men and 1.2 mg/day in women, and 2.4 µg/day for vitamin B_12_.^26^

In 2000, the Ministry of Science and Education revised the Japanese standard food composition table for the first time in 18 years,^27^ in order to reflect the diversification of Japanese dietary habits. Folate and vitamins B_6_ and B_12_ were included in the table, so that trends for these intakes could be evaluated, using the existing database of dietary surveys. Examination of these nutrient trends would be valuable for the formulation of public health recommendations.

The present study investigated the long-term trends in the dietary intake of folate and vitamins B_6_ and B_12_, according to population-based surveys in two Japanese communities between 1974 and 2001.

## METHODS

The subjects in this study are men and women aged 40 to 69 years living in the town of Ikawa, Akita prefecture, and the town of Kyowa, Ibaraki prefecture. Ikawa is located near the Sea of Japan in the north, where nearly a half of the town is forest and rice crop agriculture is the main industry. The town of Kyowa is on a plain and is located in the center of Japan, which has mainly horticultural and rice crop agriculture with other light industries.

The nutrition surveys were carried out in approximately 10% systematic samples of the participants aged 40 to 69 in the annual cardiovascular risk surveys. The participants aged over 70 were excluded from the nutrition survey because the accuracy of data based on the 24-hour dietary recall may decline with aging. For each participant in cardiovascular risk surveys, the recruitment for the nutrition surveys was made every 4 to 5 years. The subjects were not pre-informed of the recruitment for the nutrition study.

The surveys were from 1974 through 2000 for Ikawa and from 1982 through 2001 for Kyowa. All were conducted in autumns in Kyowa. In Ikawa, most of them were in springs but some data were taken in autumns from 1975 through 1981. To examine the season variations,^28^ we compared the data taken in springs and autumns and confirmed that the seasonal differences were not large enough to affect the long-term trends. The study during was stratified into seven time periods for Ikawa: 1974-1977, 1978-1981, 1982-1985, 1986-1989, 1990-1993, 1994-1997, and 1998-2000, and into four periods for Kyowa: 1982-1986, 1990-1993, 1994-1997, and 1998-2001. When there were persons who undertook the nutrition survey more than once during one survey period, we used the data in the earliest year in each survey period.

We adopted the 24-hour dietary recall method to collect the dietary data.^29^ The subjects were interviewed on what they had eaten during 24 hours before the examination. Throughout the surveys, trained dieticians carried out the interviews based on our dietary-recall manual. We continued to hire the survey dieticians for a long term in order to avoid fluctuations in interviewing technique overtime. The training sessions were conducted when we employed new dieticians. Before each survey, a meeting was held for the dieticians to follow the manual. In the interviews, actual-sized food models, pictures of food materials and dishes,^30^ and/or real foods and dishes were shown so that the subjects was easily able to recall what they had eaten. The same basic food models and the interview forms were used throughout the surveys. The intake of green tea was interviewed from 1994. As for rice and miso-soup, we asked the subjects to put the usual amount into a bowl and then we measured that quantity. We also investigated the frequencies of 18 major foods and food groups per week in order to confirm that foods in the 24-hour dietary recall were not so different from the usual foods taken. Milk and alcohol were asked as beverage in the food frequency questionnaire. Persons who had had special events such as a festival or a celebration were excluded from the surveys. The interview took approximately 30 minutes per subject.

Intakes of nutrients were estimated based on the Standard Tables of Food Composition in Japan (5th revised edition).^27^ In the surveys before the 5th edition was issued in 2000, the data were coded based on the 4th edition of Food Composition Table.^31^ We translated the data based on the 4th edition into those based on the 5th edition, using our original translation table. As for foods newly-appeared in the 5th edition, we checked the data in the latest surveys and confirmed that the newly- appeared foods were rarely taken among our subjects. It is possible that the amounts of folate and vitamins B_6_ and B_12_ contained in the same foods may change from the 1970’s to the present, but we have no data to support this possibility. Therefore, we used the data in the 5th edition throughout the surveys.

The 5th edition provides the amounts of nutrients after cooking only for selected foods.^32^^,^^33^ Thus, we evaluated all data as in conditions before cooking in order to investigate long-term trends, although there may be systematic overestimation of nutrient intakes.

We categorized 17 food groups (green/yellow and other vegetables, rice/cereal, fruits, fish/shellfish, meats, eggs, seaweed, potatoes, beans, milk/dairy products, alcohol/beverages, and others) based on the National Nutrition Survey in Japan.^34^

For primary trend analyses, we did not include dietary intake of green tea because this intake had not been interviewed in the surveys before 1994. However, for secondary analyses we estimated folate and vitamins B_6_ and B_12_ from green tea in the latest survey period, in order to examine the proportion of these nutrient intakes.

Sex- and age (10 years)- specific mean values and standard deviations of folate and vitamins B_6_ and B_12_ were calculated for each survey period as described above. Sex-specific and age-adjusted mean values and standard errors were calculated by analysis of covariance. We also evaluated intakes of major food groups that contributed to dietary intakes of these nutrients. Differences in mean values from the earliest survey period were determined using the Student’s t-test or analysis of covariance. SAS^®^ version 8.02 software (SAS Institute Inc., Cary, USA) was used for statistical analysis. P values less than 0.05 were regarded as statistical significance throughout the surveys.

## RESULTS

The number of the survey participants was between 251 and 616 for men and between 158 and 339 for women in Ikawa, and between 381 and 690 for men and between 453 and 616 for women in Kyowa. Age-adjusted mean value (standard error) of body mass index in the latest survey period was 23.9 (0.2) for men and 24.4 (0.2) for women in Ikawa, and 23.7 (0.2) and 23.5 (0.2), respectively, in Kyowa. The percentage of subjects usually taking alcohol was 88% for men and 14% for women in Ikawa, and 76% for men and 11% for women in Kyowa.

In Ikawa, age-adjusted mean intake of folate in both sexes increased approximately 30% between 1974-1977 and 1982-1985, and then leveled off (Table 1). This trend was similar for each age group. For both men and women, age-adjusted mean folate intake in 1998-2000 was 393 µg/day, which was estimated in raw food conditions and by excluding green tea. There was no secular trend in folate intake for either men or women in Kyowa. Age-adjusted mean folate intake in 1998-2000 was 332-338 µg/day for both sexes. When age was taken into account, however, mean folate intake for both sexes declined at ages 40-59, but tended to increase at ages 60-69. Thus, there was little difference overall in the mean folate intake among age groups.

**Table 1.** Trends for sex-specific mean dietary intakes of folate, vitamins B_6_, and B_12_ in Ikawa and Kyowa.

Community	Survey years	Men											Women										
	
		40-49 y.o.		50-59			60-69		total				40-49 y.o.			50-59			60-69			total	
No. of subjects																							
Ikawa																							
	1974-1977	282		209			125		616				127			110			64			301	
	1978-1981	155		219			117		491				44			38			76			158	
	1982-1985	134		156			104		394				98			104			82			284	
	1986-1989	152		101			87		340				95			93			91			279	
	1990-1993	65		110			104		279				86			131			118			335	
	1994-1997	53		102			139		294				115			99			111			325	
	1998-2000	72		87			92		251				87			109			61			257	

kyowa																							
	1982-1986	279		304			107		690				226			229			161			616	
	1990-1993	144		134			103		381				181			158			114			453	
	1994-1997	143		138			131		412				148			169			157			474	
	1998-2001	173		141			138		452				165			157			162			484	

Ikawa	Dietary intake [mean (standard deviation)]																						
Folate (µg/day)	1974-1977	332 (232)		338 (182)			320 (184)		330 (207)				314 (179)			262 (124)			250 (117)			275 (151)	
	1978-1981	399 (272)	**	323 (162)			371 (250)		364 (226)		*		311 (138)			350 (151)	**		283 (158)			315 (152)	*
	1982-1985	423 (192)	***	26 (208)	***		421 (178)	***	423 (194)		***		409 (196)	***		371 (182)	***		343 (160)	**		375 (182)	***
	1986-1989	456 (258)	***	425 (260)	**		379 (218)		420 (250)		***		363 (186)	*		384 (155)	***		347 (192)	***		365 (179)	***
	1990-1993	414 (215)	**	413 (251)	**		411 (196)	**	413 (223)		***		383 (181)	**		411 (177)	***		387 (187)	***		392 (182)	***
	1994-1997	417 (169)	*	419 (208)	**		403 (198)	**	413 (196)		***		352 (141)			405 (158)	***		385 (164)	***		381 (155)	***
	1998-2000	382 (346)		415 (210)	**		381 (225)	*	393 (261)		***		403 (215)	***		400 (202)	***		379 (152)	***		393 (196)	***

Vitamin B_6_ (mg/day)	1974-1977	1.75 (0.63)		1.67 (0.51)			1.51 (0.53)		1.64 (0.58)				1.25 (0.60)			1.12 (1.12)			1.01 (0.42)			1.13 (0.52)	
	1978-1981	1.74 (0.64)		1.61 (0.54)			1.60 (0.59)		1.65 (0.59)				1.24 (0.40)			1.23 (1.23)			0.99 (0.39)			1.15 (0.40)	
	1982-1985	1.70 (0.46)		1.63 (0.55)			1.59 (0.55)		1.64 (0.52)				1.36 (0.70)			1.26 (1.26)	*		1.07 (0.36)			1.23 (0.53)	**
	1986-1989	1.85 (0.59)		1.74 (0.62)			1.45 (0.44)		1.68 (0.59)				1.29 (0.46)			1.23 (1.23)			1.12 (0.46)			1.21 (0.44)	*
	1990-1993	1.86 (0.86)		1.72 (0.50)			1.59 (0.54)		1.72 (0.62)				1.28 (0.36)			1.30 (1.30)	**		1.20 (0.43)	*		1.26 (0.45)	***
	1994-1997	1.64 (0.59)		1.70 (0.60)			1.64 (0.51)		1.66 (0.56)				1.24 (0.42)			1.35 (1.35)	***		1.20 (0.42)	**		1.26 (0.45)	***
	1998-2000	1.55 (0.52)	**	1.68 (0.52)			1.57 (0.62)		1.60 (0.56)				1.23 (0.34)			1.33 (1.33)	***		1.24 (0.37)	**		1.27 (0.39)	***

Vitamin B_12_ (µg/day)	1974-1977	11.94 (12.79)		11.29 (10.53)			9.43 (9.38)		10.89 (11.44)				8.96 (8.96)			7.74 (7.45)			7.21 (8.15)			7.97 (8.25)	
	1978-1981	11.70 (10.63)		10.24 (9.79)			10.07 (9.68)		10.67 (10.04)				7.20 (7.20)			9.56 (7.89)			5.62 (6.44)			7.46 (6.68)	
	1982-1985	10.74 (9.82)		10.70 (9.03)			9.40 (8.94)		10.28 (9.28)				7.85 (7.85)			7.25 (6.10)			6.46 (5.04)			7.19 (6.03)	
	1986-1989	11.80 (9.01)		11.14 (12.26)			8.68 (6.66)		10.54 (9.66)				9.73 (9.73)			7.53 (5.86)			7.03 (5.30)			8.10 (7.60)	
	1990-1993	12.54 (10.96)		10.75 (9.44)			10.99 (9.44)		11.43 (9.81)				8.38 (8.38)			9.59 (8.53)	*		7.03 (6.03)			8.35 (7.41)	
	1994-1997	8.22 (8.00)	*	10.73 (9.53)			11.15 (9.00)		10.03 (9.06)				7.44 (7.44)			7.60 (6.61)			7.21 (7.90)			7.42 (7.11)	
	1998-2000	9.95 (11.80)		12.21 (9.59)			9.78 (9.16)		10.65 (10.15)				6.71 (6.71)	*		7.90 (7.31)			7.53 (6.43)			7.40 (6.70)	

Kyowa																							
Folate (µg/day)	1982-1986	394 (253)		395 (311)			281 (165)		357 (256)				381 (130)			361 (140)			317 (138)			353 (137)	
	1990-1993	360 (176)		357 (200)			307 (159)		341 (180)				323 (154)	***		368 (169)			329 (165)			340 (163)	
	1994-1997	356 (270)		348 (236)	*		335 (120)		346 (240)				335 (196)	**		352 (172)			363 (129)	**		350 (173)	
	1998-2001	324 (166)	***	345 (212)	*		328 (166)		332 (182)				303 (151)	***		373 (209)			339 (131)			338 (169)	

Vitamin B_6_ (mg/day)	1982-1986	1.68 (0.65)		1.64 (0.63)			1.31 (0.70)		1.54 (0.66)				1.29 (1.29)			1.30 (0.44)			1.17 (0.50)			1.25 (0.48)	
	1990-1993	1.55 (0.67)	*	1.62 (0.67)			1.53 (0.59)	*	1.56 (0.65)				1.26 (1.26)			1.32 (0.48)			1.22 (0.44)	*		1.26 (0.45)	
	1994-1997	1.64 (0.63)		1.67 (0.69)			1.44 (0.53)		1.58 (0.65)				1.23 (1.23)			1.28 (0.49)			1.28 (0.43)			1.26 (0.47)	
	1998-2001	1.42 (0.61)	***	1.45 (0.60)	**		1.35 (0.61)		1.41 (0.61)		***		1.17 (1.17)	*		1.22 (0.48)			1.22 (0.45)			1.20 (0.48)	

Vitamin B_12_ (µg/day)	1982-1986	8.79 (11.75)		9.32 (12.38)			5.39 (10.64)		7.83 (11.68)				6.55 (6.61)			6.53 (7.94)			5.80 (6.75)			6.29 (7.14)	
	1990-1993	9.96 (8.23)		11.06 (9.33)			9.57 (7.12)	**	10.19 (8.31)			***	6.67 (7.61)			7.67 (6.98)			6.60 (9.53)			6.98 (8.08)	
	1994-1997	7.96 (10.22)		8.91 (10.61)			7.08 (6.09)		7.98 (9.96)				6.39 (6.35)			6.67 (6.47)			7.00 (6.92)			6.69 (6.55)	
	1998-2001	8.32 (8.70)		8.18 (7.50)			6.50 (6.87)		7.67 (7.83)				5.68 (6.32)			5.79 (6.19)			6.39 (7.35)			95 (6.64)	

Symbols at the right of standard deviations represent P value for difference from the first survey; *: p<0.05, **: p<0.01, ***: p<0.001

Intake of green tea was not taken into account.

Intakes of nutrients were evaluated in conditions before cooking.

The age-adjusted mean intake of vitamin B_6_ increased approximately 10% from 1.13 mg/day in 1978-1981 to 1.27 mg/day in 1998-2000 for women in Ikawa. This trend was observed at ages 50-69, but not at ages 40-49. There was no secular trend in the mean vitamin B_6_ intake for men in Ikawa, and the age-adjusted mean value in 1998-2000 was 1.60 mg/day. Age-adjusted mean intake of vitamin B_6_ decreased 10% for men in Kyowa in the latest survey period. Age-adjusted vitamin B_6_ intake in 1998-2001 was 1.41 mg/day for men and 1.20 mg/day for women in Kyowa.

There was no secular trend in age-adjusted or age-specific mean intake of vitamin B_12_ for either sex in Ikawa. The age-adjusted mean vitamin B_12_ intake in 1998-2001 was 10.7 µg/day for men and 7.4 µg/day for women in Ikawa. There was also no secular trend in Kyowa, although mean vitamin B_12_ intakes were consistently lower than those in Ikawa. The age-adjusted mean vitamin B_12_ intake in 1998-2001 was 7.7 µg/day for men and 6.0 µg/day for women in Kyowa.

Table 2 shows the sex-specific age-adjusted mean intake by food groups in each survey period. For both sexes, the proportions of folate intake from major food sources were 38-55% from green/yellow vegetables and 13-21% from other vegetables in Ikawa, and 20-30% from green/yellow vegetables and 22-26% from other vegetables in Kyowa. The percent contributions from other food sources were 10% from rice/cereal, 3 to 10% from fruits, fish/shellfish, meats, and eggs, and less than 3% from seaweed, potatoes, beans, milk/dairy products, and alcohol/beverages, for both sexes in each community. Over 90% of the total folate intake was from the 12 food groups listed in Table 2.

**Table 2.** Trends for sex-specific age-adjusted mean dietary intakes by food group in Ikawa and Kyowa.

		Green/yellow vegetables		Other vegetables		Rice/cereal		Fruits		Fish/shellfish		Meats		Eggs		Seaweed		Potatos	Beans			Milk/dairy products		Alcohol/beverages (excluding green tea)		Alcohol/beverages (including green tea)
Folate (µg/day)																										
Men																										
Ikawa	1974-1977	123.9 (143.0)		67.4 (71.9)		42.4 (15.2)		12.0 (20.3)		17.3 (17.9)		17.3 (128.0)		11.4 (15.3)		7.6 (10.9)		6.9 (13.1)		8.3 (11.7)		2.2 (5.4)		3.2 (21.9)		
	1978-1981	145.0 (160.6)	*	64.3 (65.4)		40.6 (15.0)		16.2 (25.7)	*	16.8 (19.7)		15.5 (115.8)		12.6 (14.9)		7.3 (12.4)		9.4 (15.6)	***	12.4 (16.9)	***	3.1 (5.4)	*	2.0 (10.8)		
	1982-1985	229.6 (174.7)	***	52.3 (59.5)	***	40.8 (15.2)		16.2 (35.5)	*	15.9 (19.3)		6.3 (51.0)		15.8 (16.6)	***	9.6 (13.0)		3.5 (8.0)	***	9.6 (11.1)		3.8 (5.9)	***	5.2 (15.1)	*	
	1986-1989	192.9 (180.4)	***	56.5 (51.1)	**	40.4 (16.0)		16.1 (42.2)		15.3 (15.2)		23.9 (142.2)		16.7 (16.8)	***	6.2 (15.4)		4.7 (8.1)	**	10.5 (14.8)	*	4.6 (6.6)	***	7.1 (17.6)	**	
	1990-1993	198.7 (175.6)	***	55.3 (49.5)	**	35.5 (13.1)	***	15.5 (44.2)		16.6 (18.5)		15.6 (111.0)		14.8 (15.8)	***	3.6 (8.7)		4.7 (8.2)	*	8.6 (11.2)		5.4 (6.7)	***	9.1 (20.4)	***	
	1994-1997	210.4 (172.9)	***	50.6 (46.7)	**	33.3 (13.4)	***	13.2 (27.9)		16.6 (16.3)		6.7 (51.2)		12.8 (14.7)	**	4.9 (12.3)		6.1 (8.9)		10.1 (11.0)	*	6.4 (7.1)	***	10.3 (19.6)	***	70.7 (69.4)
	1998-2000	173.0 (168.1)	***	49.2 (52.2)	***	32.3 (13.6)	***	10.8 (22.2)		14.6 (13.6)		25.5 (176.7)		13.1 (14.6)	*	5.7 (20.6)		7.9 (11.9)		10.4 (16.0)	*	5.0 (6.9)	***	11.4 (20.8)	***	60.3 (68.5)

Kyowa	1982-1986	92.8 (144.1)		84.4 (75.4)		38.1 (15.4)		29.6 (37.9)		13.0 (13.0)		15.9 (139.4)		11.1 (14.7)		9.4 (21.0)		14.6 (23.4)		10.9 (17.6)		3.0 (5.6)		5.9 (19.7)		
	1990-1993	67.3 (92.6)	*	87.7 (75.9)		34.1 (17.4)	**	20.7 (26.7)	***	15.7 (20.1)	**	27.8 (213.1)		13.4 (15.8)		11.5 (28.3)		11.1 (17.9)		9.6 (13.8)		4.0 (6.3)	*	10.9 (25.3)	**	
	1994-1997	86.7 (124.2)		90.3 (69.2)	*	34.1 (14.8)	**	26.1 (37.4)		15.4 (18.7)	**	6.9 (60.7)		12.2 (16.2)		8.3 (17.2)		12.5 (20.6)		10.3 (21.9)		4.1 (6.4)	**	10.4 (27.4)	**	108.4 (77.0)
	1998-2001	81.6 (107.5)		81.4 (76.2)		30.0 (16.4)	***	21.0 (32.5)	**	13.5 (18.5)		8.2 (55.8)		10.9 (14.7)		10.2 (21.7)		13.1 (22.2)		13.1 (20.6)	*	4.0 (6.4)	**	14.0 (31.3)	***	101.6 (82.0)

Women																										
Ikawa	1974-1977	114.9 (123.2)		49.1 (36.9)		33.7 (12.5)		25.2 (34.1)		12.6 (13.1)		5.4 (44.2)		9.9 (13.2)		9.2 (14.3)		6.7 (10.1)		5.9 (6.5)		1.4 (3.8)		0.0 0.0		
	1978-1981	130.8 (124.3)		51.8 (43.7)		26.1 (11.5)	***	24.8 (27.1)		12.3 (18.1)		4.7 (38.8)		13.3 (16.7)	**	9.4 (14.6)		8.4 (14.0)		8.9 (13.9)	**	3.2 (5.5)	***	0.6 (7.1)		
	1982-1985	195.0 (157.4)	***	49.0 (43.4)		26.9 (9.8)	***	29.4 (38.0)		10.4 (9.7)		6.5 (45.2)		12.8 (14.7)	**	8.6 (14.4)		5.0 (12.7)		9.0 (11.9)	***	3.5 (5.1)	***	0.0 (0.0)		
	1986-1989	175.4 (148.8)	***	51.3 (46.0)		26.5 (10.8)	***	26.1 (47.7)		11.8 (11.6)		8.0 (55.9)		13.9 (16.0)	***	4.7 (9.5)		5.1 (9.2)		7.7 (9.8)	*	3.9 (5.7)	***	0.4 (3.2)		
	1990-1993	191.4 (143.0)	***	60.5 (46.0)	***	24.7 (9.7)	***	30.6 (50.2)		12.1 (14.2)		7.7 (52.4)		13.1 (13.7)	***	3.6 (10.1)		5.4 (8.5)		8.2 (8.9)	**	5.7 (6.4)	***	0.2 (1.9)		
	1994-1997	184.4 (145.6)	***	60.4 (43.0)	***	23.0 (9.5)	***	22.2 (35.6)		11.1 (9.3)		4.4 (27.3)		12.9 (15.0)	**	4.9 (9.1)		7.7 (12.8)		10.2 (11.5)	***	7.1 (7.1)	***	0.7 (5.7)	*	47.0 (55.1)
	1998-2000	195.0 (181.3)	***	58.6 (48.5)	**	5.0 (12.2)	***	23.9 (36.4)		12.1 (12.4)		5.9 (42.2)		10.2 (12.7)		4.7 (8.5)		8.9 (11.7)	*	10.3 (13.3)	***	6.6 (7.1)	***	1.2 (6.5)	**	50.0 (57.6)
																						0.0				
Kyowa	1982-1986	104.0 (118.9)		74.5 (60.8)		28.2 (13.3)		35.3 (34.4)		10.1 (10.6)		6.6 (59.5)		11.6 (14.6)		11.1 (19.2)	*	18.3 (25.9)		8.7 (11.7)		3.7 (6.0)		0.4 (3.7)		
	1990-1993	84.5 (94.7)	*	87.4 (63.2)	***	25.2 (12.4)	***	35.2 (32.0)		11.7 (12.3)		5.8 (45.9)		12.8 (13.4)		14.5 (25.0)		15.7 (23.3)		9.7 (15.1)		5.0 (6.7)	**	1.2 (6.3)		
	1994-1997	106.0 (124.4)		81.9 (55.4)	*	26.3 (14.2)		35.2 (36.2)		13.5 (16.6)	^‡^	5.7 (50.4)		10.1 (13.2)		11.2 (34.2)		14.7 (21.2)	*	9.6 (13.2)		5.7 (7.5)	***	1.1 (8.2)		87.6 (65.5)
	1998-2001	93.4 (116.3)		78.2 (57.9)		23.8 (13.5)	***	36.4 (36.7)		0.1 (12.2)		5.9 (50.6)		11.4 (15.1)		13.4 (27.9)		15.6 (23.9)		14.0 (23.0)	***	5.5 (6.5)	***	0.9 (5.1)		91.5 (71.5)

vitamin B_6_ (mg/day)																										
Men																										
Ikawa	1974-1977	0.14 (0.16)		0.15 (0.12)		0.40 (0.15)		0.05 (0.11)		0.41 (0.35)		0.11 (0.18)		0.02 (0.03)		---		0.06 (0.09)		0.04 (0.06)		0.01 (0.03)		0.23 (0.3)		
	1978-1981	0.14 (0.13)		0.12 (0.10)		0.39 (0.15)		0.07 (0.11)		0.39 (0.40)		0.13 (0.16)	*	0.02 (0.03)		---		0.07 (0.11)	**	0.07 (0.07)	***	0.02 (0.03)	*	0.20 (0.2)		
	1982-1985	0.20 (0.16)	***	0.10 (0.10)	***	0.39 (0.15)		0.07 (0.15)		0.36 (0.32)	*	0.13 (0.15)	*	0.03 (0.03)	***	---		0.03 (0.06)	***	0.05 (0.05)	*	0.02 (0.05)	***	0.20 (0.2)		
	1986-1989	0.16 (0.14)		0.13 (0.12)	***	0.38 (0.16)	*	0.07 (0.17)		0.37 (0.34)		0.14 (0.17)	*	0.03 (0.03)	***	---		0.04 (0.07)	**	0.06 (0.07)	**	0.03 (0.04)	***	0.20 (0.2)		
	1990-1993	0.15 (0.12)		0.12 (0.10)	***	0.33 (0.12)	***	0.07 (0.15)		0.41 (0.47)		0.17 (0.19)	***	0.03 (0.03)	***	---		0.04 (0.07)	*	0.05 (0.06)	*	0.03 (0.04)	***	0.20 (0.2)		
	1994-1997	0.17 (0.12)	*	0.11 (0.09)	***	0.30 (0.12)	***	0.07 (0.14)		0.41 (0.34)		0.11 (0.14)		0.02 (0.03)	**	---		0.05 (0.07)		0.07 (0.06)	***	0.04 (0.04)	***	0.20 (0.2)		0.22 (0.2)
	1998-2001	0.16 (0.13)		0.11 (0.12)	***	0.27 (0.12)	***	0.06 (0.16)		0.35 (0.32)		0.15 (0.21)	***	0.02 (0.03)	*	---		0.07 (0.10)		0.07 (0.06)	***	0.03 (0.04)	***	0.19 (0.2)		0.21 (0.2)

Kyowa	1982-1986	0.10 (0.13)		0.16 (0.13)		0.33 (0.13)		0.10 (0.15)		0.37 (0.42)		0.11 (0.18)		0.02 (0.03)		---		0.09 (0.14)		0.05 (0.07)		0.02 (0.03)		0.13 (0.2)		
	1990-1993	0.08 (0.10)		0.17 (0.16)		0.27 (0.12)	***	0.07 (0.10)	**	0.41 (0.44)	*	0.14 (0.21)	**	0.02 (0.03)		---		0.07 (0.11)		0.05 (0.06)		0.02 (0.04)	*	0.16 (0.2)		
	1994-1997	0.11 (0.13)		0.17 (0.13)	*	0.27 (0.11)	***	0.09 (0.15)		0.38 (0.43)		0.13 (0.19)	*	0.02 (0.03)		---		0.08 (0.13)		0.06 (0.07)		0.02 (0.04)	**	0.15 (0.2)		0.21 (0.2)
	1998-2001	0.10 (0.12)		0.16 (0.15)		0.21 (0.11)	***	0.08 (0.14)		0.31 (0.37)		0.11 (0.16)		0.02 (0.03)		---		0.09 (0.15)		0.06 (0.07)		0.02 (0.03)	*	0.15 (0.2)		0.20 (0.2)

Women																										
Ikawa	1974-1977	0.14 (0.23)		0.13 (0.09)		0.31 (0.12)		0.12 (0.19)		0.25 (0.25)		0.05 (0.10)		0.02 (0.03)		---		0.06 (0.08)		0.03 (0.03)		0.01 (0.02)		0.00 (0.0)		
	1978-1981	0.13 (0.13)		0.13 (0.10)		0.24 (0.09)	***	0.11 (0.14)		0.22 (0.20)		0.07 (0.11)		0.02 (0.03)	**	---		0.07 (0.10)		0.05 (0.06)	**	0.02 (0.03)	**	0.01 (0.1)		
	1982-1985	0.18 (0.13)	**	0.13 (0.21)		0.25 (0.10)	***	0.15 (0.25)		0.24 (0.19)		0.08 (0.12)	**	0.02 (0.03)	*	---		0.04 (0.08)	*	0.05 (0.05)	***	0.02 (0.03)	***	0.00 (0.0)		
	1986-1989	0.15 (0.12)		0.12 (0.10)		0.23 (0.08)	***	0.13 (0.22)		0.24 (0.20)		0.10 (0.12)	***	0.03 (0.03)	***	---		0.04 (0.07)		0.04 (0.04)		0.02 (0.03)	***	0.01 (0.0)		
	1990-1993	0.16 (0.11)		0.14 (0.09)	*	0.21 (0.07)	***	0.12 (0.21)		0.26 (0.23)		0.10 (0.13)	***	0.02 (0.03)	**	---		0.05 (0.07)		0.05 (0.05)	***	0.03 (0.04)	***	0.00 (0.0)		
	1994-1997	0.17 (0.12)	*	0.14 (0.10)	*	0.18 (0.07)	***	0.11 (0.16)		0.25 (0.21)		0.10 (0.12)	***	0.02 (0.03)	**	---		0.06 (0.09)		0.06 (0.05)	***	0.04 (0.04)	***	0.01 (0.1)	*	0.03 (0.1)
	1998-2000	0.19 (0.15)	***	0.12 (0.08)		0.19 (0.08)	***	0.10 (0.15)		0.26 (0.23)		0.10 (0.13)	***	0.02 (0.02)		---		0.07 (0.09)	**	0.10 (0.49)	***	0.04 (0.04)	***	0.01 (0.1)	*	0.04 (0.1)

Kyowa	1982-1986	0.11 (0.10)		0.15 (0.13)		0.22 (0.09)		0.11 (0.12)		0.23 (0.26)		0.08 (0.12)		0.02 (0.03)		---		0.12 (0.16)		0.04 (0.05)		0.02 (0.03)		0.00 (0.0)		
	1990-1993	0.11 (0.11)		0.17 (0.13)		0.19 (0.07)	***	0.12 (0.12)		0.26 (0.29)		0.10 (0.13)	*	0.02 (0.03)		---		0.10 (0.14)		0.05 (0.06)		0.03 (0.04)	*	0.01 (0.1)		
	1994-1997	0.13 (0.13)	**	0.17 (0.12)		0.19 (0.09)	***	0.13 (0.15)		0.24 (0.26)		0.11 (0.13)	***	0.02 (0.02)		---		0.10 (0.13)	*	0.05 (0.05)		0.03 (0.04)	***	0.01 (0.1)	*	0.07 (0.1)
	1998-2001	0.13 (0.14)	*	0.16 (0.12)		0.15 (0.07)	***	0.14 (0.16)	**	0.20 (0.25)	*	.10 (0.14)	**	0.02 (0.03)		---		0.10 (0.14)		0.06 (0.06)	***	0.03 (0.04)	***	0.01 (0.0)	*	0.07 (0.1)

vitamin B_12_ (µg/day)																										
Men																										
Ikawa	1974-1977	---		---		---		---		9.85 (10.10)		0.86 (5.70)		0.24 (0.32)		0.07 (0.37)		---		---		0.13 (0.32)		0.05 (0.3)		
	1978-1981	---		---		---		---		9.15 (9.41)		0.90 (4.14)		0.26 (0.31)		0.10 (0.62)		---		---		0.20 (0.36)	**	0.03 (0.2)		
	1982-1985	---		---		---		---		9.07 (9.22)		0.54 (1.97)		0.33 (0.35)	***	0.08 (0.38)		---		---		0.24 (0.37)	***	0.07 (0.2)	*	
	1986-1989	---		---		---		---		8.77 (7.86)		1.13 (4.84)		0.36 (0.36)	***	0.09 (0.33)		---		---		0.28 (0.39)	***	0.10 (0.3)	**	
	1990-1993	---		---		---		---		9.41 (9.30)		0.93 (3.92)		0.31 (0.33)	***	0.06 (0.26)		---		---		0.35 (0.42)	***	0.13 (0.3)	***	
	1994-1997	---		---		---		---		8.56 (8.21)		0.86 (3.93)		0.27 (0.31)	**	0.12 (0.42)		---		---		0.41 (0.43)	***	0.15 (0.3)	***	0.15 (0.3)
	1998-2001	---		---		---		---		8.28 (7.81)		1.41 (6.72)	*	0.28 (0.32)	*	0.12 (0.56)		---		---		0.35 (0.44)	***	0.16 (0.3)	***	0.16 (0.3)

Kyowa	1982-1986	---		---		---		---		7.00 (9.14)		0.69 (4.39)		0.24 (0.32)		0.22 (0.66)		---		---		0.19 (0.35)		0.08 (0.3)		
	1990-1993	---		---		---		---		7.91 (8.90)	**	1.34 (8.00)	*	0.28 (0.33)		0.26 (0.85)		---		---		0.25 (0.39)	*	0.16 (0.4)	**	
	1994-1997	---		---		---		---		6.62 (7.83)		0.42 (2.05)		0.26 (0.36)		0.21 (0.55)		---		---		0.27 (0.42)	**	0.15 (0.4)	**	0.15 (0.4)
	1998-2001	---		---		---		---		6.20 (7.56)		0.50 (1.97)		0.24 (0.35)		0.27 (0.67)		---		---		0.25 (0.40)	**	0.20 (0.5)	***	0.20 (0.5)

Women																										
Ikawa	1974-1977	---		---		---		---		7.40 (8.02)		0.27 (1.44)		0.21 (0.29)		0.14 (0.71)		---		---		0.09 (0.24)		0.00 (0.0)		
	1978-1981	---		---		---		---		6.12 (6.67)		0.28 (1.27)		0.28 (0.35)	**	0.10 (0.30)		---		---		0.20 (0.33)	***	0.01 (0.1)		
	1982-1985	---		---		---		---		6.21 (5.86)		0.42 (1.71)		0.27 (0.31)	**	0.07 (0.23)		---		---		0.22 (0.30)	***	0.00 (0.0)		
	1986-1989	---		---		---		---		6.95 (7.20)		0.49 (2.28)		0.29 (0.34)	***	0.07 (0.29)		---		---		0.25 (0.36)	***	0.01 (0.1)		
	1990-1993	---		---		---		---		7.12 (7.20)		0.44 (1.94)		0.28 (0.29)	***	0.06 (0.30)		---		---		0.37 (0.40)	***	0.00 (0.0)		
	1994-1997	---		---		---		---		6.15 (6.58)	*	0.33 (1.41)		0.28 (0.32)	**	0.12 (0.31)		---		---		0.44 (0.40)	***	0.01 (0.1)	*	0.01 (0.1)
	1998-2000	---		---		---		---		6.15 (6.49)		0.35 (1.49)		0.22 (0.27)		0.10 (0.28)		---		---		0.44 (0.43)	***	0.02 (0.1)	**	0.02 (0.1)

Kyowa	1982-1986	---		---		---		---		5.13 (6.26)		0.38 (2.09)		0.25 (0.33)		0.26 (0.57)		---		---		0.23 (0.37)		0.01 (0.1)		
	1990-1993	---		---		---		---		5.56 (6.80)		0.39 (2.09)		0.27 (0.28)		0.36 (0.78)		---		---		0.31 (0.41)		0.02 (0.1)		
	1994-1997	---		---		---		---		5.41 (7.83)		0.33 (1.67)		0.21 (0.28)		0.30 (1.06)		---		---		0.35 (0.44)		0.02 (0.1)		0.02 (0.1)
	1998-2001	---		---		---		---		4.56 (6.32)		0.37 (1.74)		0.26 (0.40)		0.35 (0.82)		---		---		0.34 (0.41)		0.01 (0.1)		0.01 (0.1)

Symbols at the right of standard deviations represent P value for difference from the first survey; *: p<0.05, **: p<0.01, ***: p<0.001

For alcohol/beverages, nutrient inatekes were shown when we excluded or included green tea.

Intakes of nutrients were evaluated in conditions before cooking.

**Figure 1.**
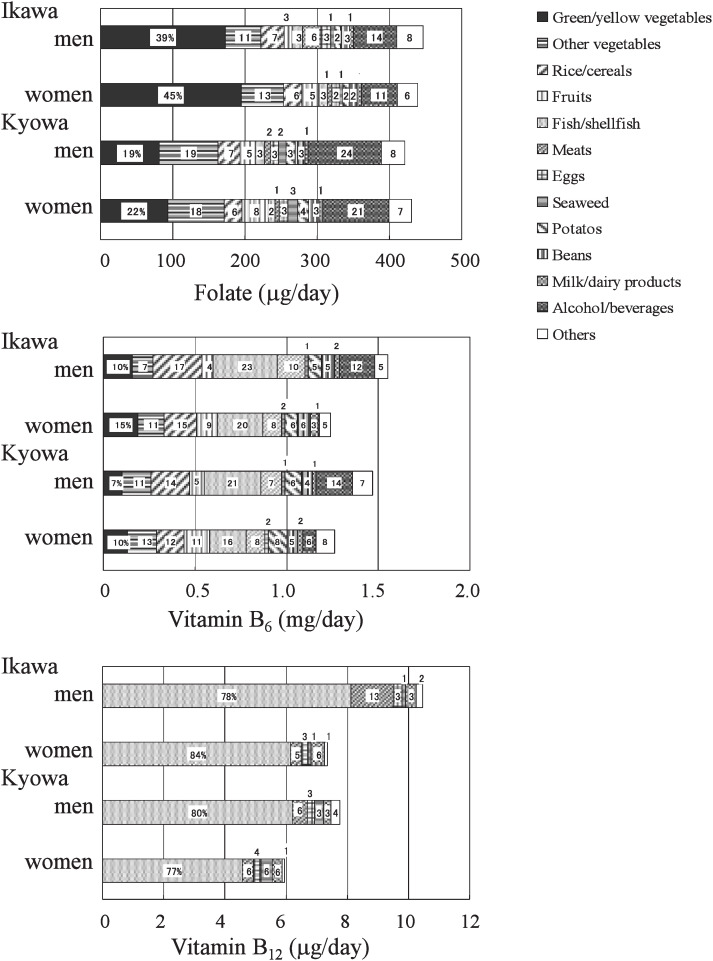
Sex-specific age-adjusted mean intakes of folate, vitamin B_6_, and vitamin B_12_ by food group including green tea as alcohol/beverages in 1998-2000 for Ikawa and in 1998-2001 for Kyowa.

When we took green tea into account in the latest survey, the proportion of folate intake from alcohol/beverages was 14% for Ikawa men, 11% for Ikawa women, 24% for Kyowa men, and 21% for Kyowa women, while those excluding green tea were 3%, 0.3%, 4%, and 0.3%, respectively. The proportions of folate intake by food group in the latest survey period are shown in Figure 1. Mean folate intake from alcohol/beverages including green tea in the latest survey period was 60.3 µg/day for men and 50.0 µg/day for women in Ikawa, and 101.6 µg/day for men and 91.5 µg/day for women in Kyowa.

In Ikawa, mean folate intake for both sexes from green/yellow vegetables doubled from 1974-1977 through 1982-1985, and then leveled off. Spinach mainly contributed to this increase, which comprised 55-82 % of total green/yellow vegetables in men and 50-75 % in women. Folate intake from spinach was 68 µg/day for men and 57 µg/day for women in 1974-1977, which increased three-fold to 186 and 146 µg/day for men and women, respectively, in 1982-1985. In Kyowa, folate intake from green/yellow vegetables decreased between 1982-1986 and 1990-1993. This was mainly due to the decreased intake from spinach from 71.7 to 29.0 µg/day for men and from 79.5 to 37.5 µg/day for women. The folate intake from broccoli slightly increased (2.9 µg/day in men and 7.8 µg/day in women). In 1998-2001, there was a higher folate intake from spinach in Ikawa (142 µg/day for men and 136 µg/day for women) than in Kyowa (47 µg/day for men and 38 µg/day for women). Mean folate intake from eggs, milk/dairy products, and beans increased in a similar manner, whereas that from rice/cereal decreased for both sexes. Mean folate intake from other vegetables declined for men but increased for women. In Kyowa, the mean folate intake from beans and milk/dairy products increased, whereas that from rice/cereal decreased for both sexes.

The proportions of vitamin B_6_ intake from major food sources were 16-23% from fish/shellfish, 12-17% from rice/cereal, 7-15% from green/yellow vegetables, 7-13% from other vegetables, 7-10% from meats, 5-8% from potatoes, 4-6% from beans, and 1-2% from eggs and milk/dairy products, for both sexes in each community (Figure 1). There was a large sex-difference in the percent contribution from alcohol/beverages and fruits in both communities. The proportion of vitamin B_6_ intake from alcohol/beverages was 9-15% for men and 0.1-0.9% for women, and that from fruits was 3-6% for men and 8-13% from women. Ninety percent of the total vitamin B_6_ intake was from the above 11 food groups. In the vitamin B_6_ intake from alcohol/beverage, beer and sake were predominant sources. Ninety-nine percent of that intake from alcohol/beverage was from beer and sake (0.15-0.20mg for men in the latest survey period). Mean vitamin B_6_ intake from rice/cereal gradually decreased for both sexes in each community, whereas intake from vegetables, meats, beans, and milk/daily products increased except for that in Kyowa men.

The proportions of vitamin B_12_ intake from major food sources were 77-84% from fish/shellfish, 5-13% from meats, and 3-6% from milk/daily products, 1-6% from seaweed, and 3-4% from eggs, for both sexes in each community (Figure 1). Almost 100% of vitamin B_12_ intake was from the above five food groups. In Ikawa, the proportion of vitamin B_12_ intake from fish/shellfish tended to decrease from 88% in 1974-1977 to 76% in 1998-2001 for men and 91% in 1974-1977 to 84% in 1998-2001 for women.

Food specific percentages of total intakes are in the bar graphs. Intake is evaluated in conditions before cooking.

## DISCUSSION

In the present long-term nutrition study, we found increased folate intake in the mid-1980’s due to increased vegetables intake in Ikawa men and women, and increased vitamin B_6_ intake in the 1980’s due to increased intake of vegetables, meats, beans and milk/dairy products for Ikawa women. Secular trends were not found for vitamin B_6_ or B_12_ intakes.

For both sexes in each community, the largest contributor for these nutrients was total vegetables (38-58% of total intake) for folate, fish/shellfish (16-23%) for vitamin B_6_, and fish/shellfish (78-84%) for vitamin B_12_. Alcohol/beverages including green tea was the second largest source for folate intake (11-24%).

The increase in folate intake in the mid-1980’s in Ikawa was largely due to an increase in green/yellow vegetables consumption. Green/yellow vegetables were largely comprised of spinach (55-82% in men and 50-75% in women in the survey periods), which contains 210 µg of folate per 100 g. Folate intake from spinach was 68 µg/day for men and 57 µg/day for women in 1974-1977, which increased three-fold to 186 µg/day and 146 µg/day for men and women, respectively, in 1982-1985. This increase was nearly equal to the increase in total folate intake over time. In Kyowa, however, folate intake from green/yellow vegetables decreased between 1982-1986 and 1990-1993, which was mainly due to the decreased intake of spinach. The folate intake from broccoli increased to some degree, but this increase was not large enough to compensate for the decreased folate intake from spinach. Mean folate intake was about 15% higher in Ikawa than in Kyowa for both sexes in 1998-2001. This community difference was mainly due to a three-fold higher folate intake from spinach in Ikawa than in Kyowa. In Kyowa, the mean folate intake in men and women aged 40-49 decreased between 1982-1986 and 1998-2001. This was mainly due to the decreased intake from green/yellow vegetables for women and other vegetables for men.

Consumption of green tea, which contained 32 µg of folate per cup, contributed 11-13% of total folate intake in Ikawa, and 21-24% of total folate intake in Kyowa. This result indicates that green tea is the third most important contributor to folate intake next to green/yellow vegetables and other vegetables.^35^ The participants of this survey drank an average of 1.5 cups of green tea per day in Ikawa and 2.8 cups in Kyowa for both sexes.

We did not find any secular trends in mean intakes of vitamin B_6_ or B_12_ except for B_6_ in Ikawa women and in Kyowa men. The stable intake of vitamin B_6_ was counter-balanced by a decreased intake from rice/cereals and an increased intake from vegetables, meats, beans, and milk/daily products. There was an increased mean intake of vitamin B_6_ in Kyowa women, which was due to an additional increase in intake of green/yellow vegetables. A decreased mean intake of vitamin B_6_ in Kyowa men was due to the reduced intake of seafood as well as rice/cereals. The stable intake of vitamin B_12_ was due to a stable intake of fish/shellfish, which is a predominant source of vitamin B_12_. Mean vitamin B_12_ intake was approximately 20% higher in Ikawa than in Kyowa for both sexes during 1998-2001. This community difference was due mostly to a higher intake of fish/shellfish. The higher intake of seafood in Ikawa is explained by its geographical location near the Sea of Japan.

Although the mean vitamin B_6_ intake did not differ between the communities, the mean intake was approximately 20% higher for men than for women in each community. This sex difference was largely due to higher intakes of seafood and beer and sake.

Although there was no significant secular trend in mean folate intake from meats, there were large variations for men in both communities. This large variation was due to liver, which contains a large amount of folate (810-1300 µg per 100 g or 255µg per portion size of 55g) compared with green/yellow vegetables (approximately 100-200 µg per 100 g). When we excluded liver in calculating mean folate intake from meats, the age-adjusted mean value was smaller, ranging between 2.7 and 4.2 µg/day for men in Ikawa and between 2.0 and 3.2 µg/day for men in Kyowa. Although liver caused the large variations in folate intake from meats, its influence on the total intake was small because liver was rarely eaten.

The declines in mean vitamin B_6_ intake for Kyowa men and in mean folate intake for Kyowa men and women aged 40-59 years could potentially cause nutritional problems. The decline in vitamin B_6_ intake was due to decreased intakes of both seafood and rice/cereals. The decline in folate intake among young age groups was due to decreased intake of vegetables and rice/cereals.

In our study, nutrient intakes were evaluated in raw food conditions because there was no systematic database about nutrient loss by cooking. Estimated changes of nutrients after cooking are 79 % for folate, 75 % for vitamin B_6_, and 97 % for vitamin B_12_.^33^ Thus, dietary intakes of folate and vitamin B6 may be overestimeated systematically in the present study.

In summary, we investigated long-term trends in dietary intakes of folate and vitamins B_6_, and B_12_ among Japanese adults in two rural communities, and found that there was an increase in the dietary intake of folate for men and women between the 1970’s and the 1980’s, along with the increased intake of green/yellow vegetables. Folate intake was determined mainly by vegetables, especially spinach, while intakes of vitamins B_6_ and B_12_ were by fish/shellfish.
